# Mandatory containment of COVID-19 patients in Monastir: Legislative framework and impact on freedoms

**DOI:** 10.1192/j.eurpsy.2022.1266

**Published:** 2022-09-01

**Authors:** I. Betbout, B. Amemou, A. Ben Haouala, S. Iben Khedher, M. Benzarti, F. Zaafrane, A. Mhalla, L. Gaha

**Affiliations:** Fattouma Bourguiba Hospital, Psychiatry, Psychiatry Research Laboratory Lr05es10 “vulnerability To Psychoses” Faculty Of Medicine Of Monastir, University Of Monastir, monastir, Tunisia

## Abstract

**Introduction:**

Tunisia found itself in an exceptional situation during the covid 19 pandemic requiring a legal regime of exceptionality and sanitary necessity with a double challenge: the fight against the sanitary crisis, and the preservation of democratic gains

**Objectives:**

To describe the legislative framework put in place concerning patients with COVID-19 who stayed at the compulsory containment and to discuss the legality of these emergency decisions

**Methods:**

The authors conducted a descriptive cross-sectional study of patients with COVID-19 staying in the compulsory containment centre of Monastir, with a review of the literature The data were collected through telephone calls. A review of the literature as well as a consultation of the different legislative

**Results:**

The average age was 41.39 ± 1.26 and the sex ratio was 1.17.Imported cases represented 45.3% of the sample and 23% of them expressed a desire to consult a specialist. The duration of mandatory confinement was on average 35.86±1.31 days with extremes ranging from 7 to 86 days. Concerning the legislative framework of the emergency decisions taken during the first wave, the President of the Republic and the Head of Government used Articles 80 and 70 of the Tunisian Constitution, respectively, to issue legislative texts announcing the state of emergency and accompanying. Thus, these legislative measures were restrictive of rights and freedoms and seriously threatened the fragile gains of our democracy

**Conclusions:**

COVID-19 redefined not only the health system but also the economic conditions, as well as the normative and legislative system 2014

**Disclosure:**

No significant relationships.

